# Predictive Value of Preoperative CT Findings for Bowel Ischemia in Patients with Blunt Mesenteric Injury: A Retrospective Cohort Study

**DOI:** 10.3390/jcm15041330

**Published:** 2026-02-07

**Authors:** Suyeong Hwang, Sung Hoon Cho, Chang-Yeon Jung, Kyoung Hoon Lim, Gun Woo Kim

**Affiliations:** Department of Surgery, Trauma Center, Kyungpook National University Hospital, School of Medicine, Kyungpook National University, Daegu 41944, Republic of Korea; tndud4857@knu.ac.kr (S.H.); chossis@knu.ac.kr (S.H.C.); gsjcygs@gmail.com (C.-Y.J.); drlimkh@knu.ac.kr (K.H.L.)

**Keywords:** blunt abdominal trauma, mesenteric injury, bowel ischemia, computed tomography, nonoperative management

## Abstract

**Background:** Delayed bowel ischemia is a major cause of failure of nonoperative management in patients with blunt mesenteric injury. Although decreased bowel wall enhancement on computed tomography (CT) is a definitive sign of bowel ischemia, it is uncommon and may be absent on early imaging. This study aimed to identify specific CT findings that predict bowel ischemia to distinguish patients requiring surgery from those suitable for conservative management. **Methods:** We retrospectively reviewed 174 patients with blunt mesenteric injury treated at a Level 1 trauma center between January 2013 and December 2024. Initial CT findings were classified as mesenteric contrast extravasation freely extending into the peritoneal cavity (extravasation type 1), contrast extravasation tracking along the bowel contour (extravasation type 2), pseudoaneurysm, mesenteric haziness, mesenteric hematoma, interloop fluid, dependent portion fluid, and decreased bowel wall enhancement. Predictors of bowel ischemia were evaluated using univariate analysis and ridge-penalized multivariable logistic regression. **Results:** Bowel ischemia occurred in 30 patients (17.2%). Decreased bowel wall enhancement was rare (4.6%) but demonstrated perfect specificity and positive predictive value (both 100%), with low sensitivity (26.7%). Extravasation type 2 showed high specificity (97.2%) and remained an independent predictor of bowel ischemia. Dependent portion fluid showed relatively high sensitivity, whereas mesenteric haziness and mesenteric hematoma were inversely associated with ischemia. **Conclusions:** Contrast extravasation tracking along the bowel contour and decreased bowel wall enhancement on early CT are strong predictors of bowel ischemia in patients with blunt mesenteric injury. These findings should prompt consideration of early surgical exploration, even in patients who initially appear hemodynamically stable.

## 1. Introduction

In blunt abdominal trauma, hollow viscus and mesenteric injuries are reported in approximately one to three percent of patients [1,2,3]. Although hollow viscus injuries are relatively uncommon, their diagnostic difficulty and the high morbidity associated with delayed diagnosis and treatment make them a critical concern in trauma care [4,5,6].

In contemporary trauma practice, E-FAST and CT play central roles in evaluating and managing blunt abdominal trauma [2,4,7,8]. Exploratory surgery is indicated when CT demonstrates free intraperitoneal air, findings suggestive of bowel ischemia, or when signs of peritonitis are present on physical examination despite negative CT findings. Conversely, when CT reveals only nonspecific findings—such as fluid collection presumed to result from bleeding—and the patient remains hemodynamically stable without peritonitis signs, conservative management may be appropriate. In line with recent World Society of Emergency Surgery (WSES) guidelines, there has been an increasing trend toward nonoperative management (NOM) in carefully selected patients with blunt bowel and mesenteric injuries who are hemodynamically stable and lack definitive indications for immediate surgery. In patients with blunt mesenteric injury undergoing NOM, bowel ischemic necrosis secondary to compromised mesenteric perfusion represents the most serious and clinically significant complication. Moreover, although decreased bowel wall enhancement on CT is a definitive sign, it is rare [9] and may not be visible on initial CT obtained soon after injury because of delayed manifestation of ischemic changes. Given these limitations, early identification of patients at risk for delayed bowel ischemia remains a major clinical challenge [10,11]. Most previous studies have primarily focused on identifying bowel and mesenteric injuries requiring immediate surgical intervention, rather than predicting the subsequent development of bowel ischemia in initially stable patients. This study was therefore designed to identify CT findings on initial imaging of patients with mesenteric injury that may predict delayed bowel ischemia beyond decreased bowel wall enhancement, to improve the ability to distinguish patients suitable for conservative management from those requiring surgical intervention.

## 2. Methods

### 2.1. Patients and Study Design

We retrospectively reviewed 174 patients with mesenteric injury due to blunt trauma who were treated at the Level 1 Trauma Center of Kyungpook National University Hospital between January 2013 and December 2024. Throughout this 11-year inclusion period, although CT scanner technology and hardware were updated, our institutional trauma management protocols and CT acquisition parameters remained strictly standardized to ensure consistency in patient care and image evaluation. Patients were eligible for inclusion if they had sustained blunt abdominal trauma and demonstrated evidence of mesenteric injury on contrast-enhanced CT, defined as mesenteric abnormalities (such as mesenteric haziness or hematoma) or unexplained intraperitoneal fluid in the absence of identifiable solid-organ injury.

Exclusion criteria included penetrating injury, bowel perforation, a delay of more than 3 h from trauma to initial CT, absence of contrast-enhanced CT, major solid-organ injury (spleen, liver, pancreas, or kidney), and age younger than 17 years.

Mesenteric injury was defined as either CT findings suggestive of mesenteric injury, such as mesenteric haziness or hematoma, or unexplained intraperitoneal fluid collection in the absence of identifiable solid-organ injury. All CT findings were independently reviewed by one board-certified radiologist and one trauma surgeon. Abdominal explorations were performed by seven attending trauma surgeons, and bowel ischemia was confirmed intraoperatively.

Patients were categorized into bowel ischemia and non-ischemia groups. CT findings were then compared between groups to assess the predictive value of each variable for bowel ischemia.

### 2.2. Definition of CT Findings

**Figure 1:**
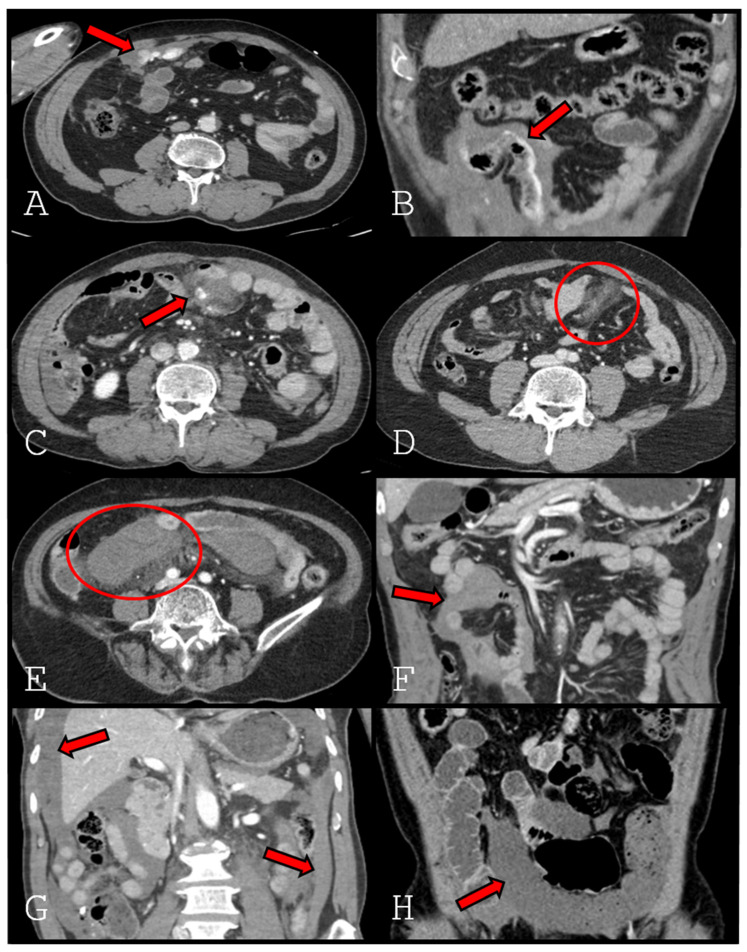
Representative CT findings in mesenteric injury after blunt trauma. (**A**). Extravasation type 1 (arrow). (**B**). Extravasation type 2 (arrow). (**C**). Pseudoaneurysm (arrow). (**D**). Mesenteric haziness (circle). (**E**). Mesenteric hematoma (circle). (**F**). Interloop fluid (arrow). (**G**). Dependent portion fluid (arrow). (**H**). Decreased bowel wall enhancement (arrow).

All CT findings were assessed on portal venous–phase images obtained from contrast-enhanced abdominopelvic CT. In principle, our institutional protocol for initial abdominopelvic trauma CT excludes oral contrast and consists of sequential non-contrast and portal venous phase scans, unless specifically indicated otherwise. The following CT findings were evaluated and defined (Figure 1).

Extravasation type 1: Active contrast extravasation from the mesentery and extending freely into the peritoneal cavity, indicating intraperitoneal bleeding.

Extravasation type 2: Active contrast extravasation originating from the mesentery and extending into the peritoneal cavity, tracking along the bowel contour.

Pseudoaneurysm: A well-circumscribed, contrast-enhancing sac contiguous with a mesenteric artery, or active contrast extravasation confined within the mesentery without clear extension into the peritoneal cavity.

Mesenteric haziness: Ill-defined increased attenuation or stranding of mesenteric fat.

Mesenteric hematoma: A well-defined or localized hyperattenuating collection within the mesentery, distinct from adjacent bowel or vessels.

Interloop fluid: A fluid collection located between adjacent bowel loops.

Dependent portion fluid: Large free-fluid accumulation layering in dependent portions of the peritoneal cavity, including the pelvis, paracolic gutters, hepatic dome, Morrison pouch, and splenic bed.

Decreased bowel wall enhancement: Segmental or focal reduction in bowel wall enhancement compared with adjacent normal bowel loops [12,13].

All CT images were independently reviewed by a radiologist and a trauma surgeon. The reproducibility of ‘extravasation type 2′ was specifically assessed; in cases of initial discrepancy, a consensus was reached through a joint review of the anatomical tracking pattern. This standardized approach was employed to minimize interobserver variability and ensure the reliability of the classification.

### 2.3. Indications for Emergency Operation

Patients who were hemodynamically unstable at presentation or who showed only a transient response after fluid or transfusion resuscitation underwent immediate emergency surgery. In contrast, patients who were initially hemodynamically stable and managed conservatively but later became unstable or demonstrated suspected bowel ischemia on follow-up CT underwent delayed surgery.

### 2.4. Statistical Analysis

The primary endpoint was bowel ischemia (present/absent). Univariate analyses were performed using Fisher’s exact test, with sensitivity, specificity, positive predictive value (PPV), negative predictive value (NPV), and odds ratios (ORs) reported with 95% confidence intervals (CIs). Multivariable modeling employed ridge-penalized logistic regression including all prespecified CT covariates, with the regularization parameter (λ) selected by five-fold cross-validation. Statistical significance was defined as two-sided α = 0.05, without multiplicity adjustment. Descriptive statistics and contingency-table analyses were conducted in PASW Statistics for Windows, version 18.0 (SPSS Inc., Chicago, IL, USA). Penalized regression models were fitted using R, version 4.3.2 (glmnet 4.1–8). An AI-assisted coding workflow (ChatGPT, GPT-4) was used to draft and refine analysis scripts, which were subsequently verified against SPSS and R outputs.

## 3. Results

**Figure 2:**
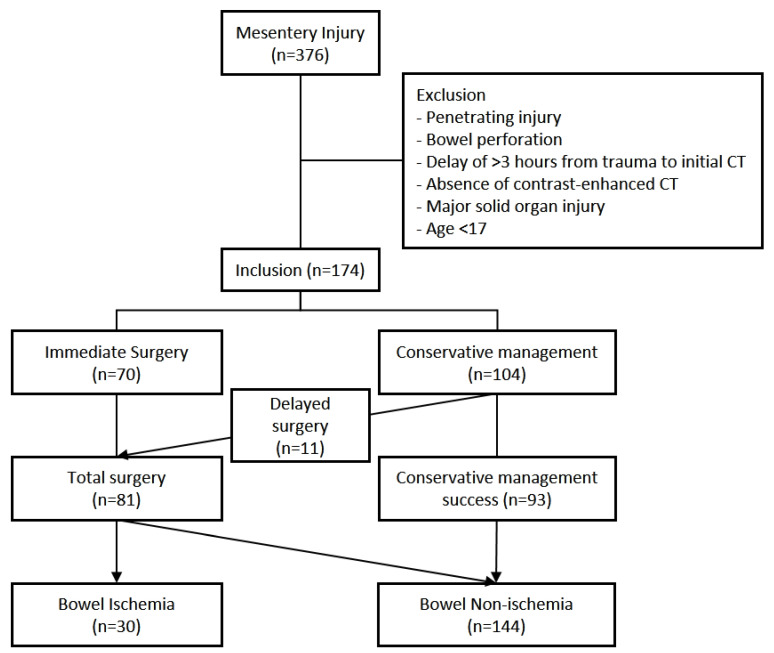
Inclusion and exclusion criteria of mesentery injury.

From January 2013 to December 2024, a total of 376 patients presented to our trauma center with mesenteric injury. Of these, 15 patients younger than 17 years, 48 with penetrating injury, 82 with bowel perforation, 48 with major solid organ injury, 6 who underwent initial CT more than 3 h after injury, and 3 without initial CT were excluded. The remaining 174 patients constituted the study cohort (Figure 2).

Baseline characteristics are summarized in Table 1. Of the 174 patients, 138 were male and 36 were female. The mean age was 53.7 ± 15.6 years, the mean Injury Severity Score (ISS) was 13.8 ± 8.7 and the mean shock index at presentation was 0.82 ± 0.30. Mechanisms of injury included in-car traffic accidents (73 patients), motorcycle accidents (27 patients), pedestrian trauma (20 patients), crush injuries (32 patients), falls (12 patients), slip-down injuries (5 patients), and bicycle accidents (5 patients). Age, sex distribution, shock index, and 24 h transfusion volume did not differ significantly between groups. The ischemia group had a significantly higher ISS compared with the non-ischemia group (18.5 ± 9.2 vs. 12.8 ± 8.3, *p* = 0.001). Pedestrian trauma was absent in the ischemia group (0% vs. 13.9%, *p* = 0.027), whereas the proportions of other mechanisms were similar between groups.

Among the study cohort, 70 patients underwent immediate surgery for bleeding or suspected bowel injury, while 104 were initially managed conservatively. Of those managed conservatively, 5 required operative intervention for persistent bleeding, and 6 underwent delayed surgery due to bowel ischemia identified on follow-up CT. Overall, bowel ischemia was intraoperatively confirmed in 30 patients (17.2%). In addition, 11 patients required delayed surgery during conservative management, resulting in a conservative management failure rate of 10.6%.

Decreased bowel wall enhancement (8/174, 4.6%) was rare but demonstrated perfect specificity and PPV (both 100%), with a sensitivity of 26.7%, and showed a notable potential association with bowel ischemia (OR 109.18; 95% CI, 6.09–1957.81; *p* < 0.001). However, given the wide confidence interval, this finding should be interpreted as an adjunctive indicator rather than a definitive predictor. Extravasation type 2 (contrast tracking along the bowel contour) also demonstrated high specificity (97.2%) and PPV (63.6%) with a significant association (OR 9.96; 95% CI, 2.86–34.70; *p* < 0.001). Dependent portion fluid exhibited relatively high sensitivity (70.0%) with a modest but significant association (OR 2.60; 95% CI, 1.13–5.96; *p* = 0.026), supporting its utility in excluding ischemia. In contrast, mesenteric haziness (OR 0.25; 95% CI, 0.10–0.68; *p* = 0.004) and mesenteric hematoma (OR 0.21; 95% CI, 0.07–0.68; *p* = 0.003) showed inverse associations with bowel ischemia (Table 2). In multivariable analysis, decreased bowel wall enhancement (aOR ≈ 5.5; 95% CI ≈ 2.6–9.4) and extravasation type 2 (aOR ≈ 3.1; 95% CI ≈ 1.5–5.7) remained independent predictors (Table 3).

## 4. Discussion

In this study, we evaluated the predictive value of preoperative CT findings for bowel ischemia in patients with mesenteric injury after blunt trauma. Although NOM is often feasible for stable patients, identifying those at risk for delayed bowel ischemia remains a critical diagnostic challenge. Our results demonstrate that decreased bowel wall enhancement and contrast extravasation along the bowel contour (Extravasation Type 2) are strong independent predictors of bowel ischemia. In contrast, mesenteric haziness and mesenteric hematoma are inversely associated with ischemia, suggesting a lower likelihood of ischemia.

**Figure 3:**
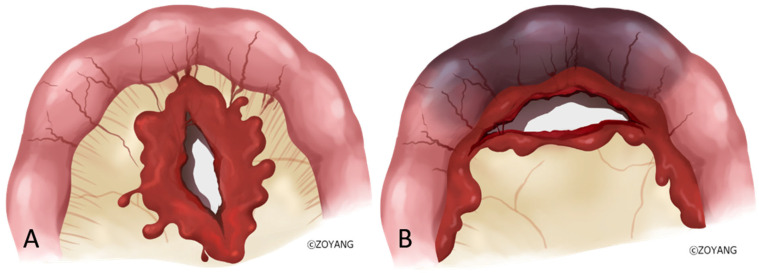
Proximal and distal mesenteric injuries: different bleeding patterns and implications for bowel ischemia. (**A**). Proximal mesenteric injury with maintained distal perfusion and a low likelihood of bowel ischemia. (**B**). Distal mesenteric injury serving as a predictor of subsequent segmental bowel ischemia. All images are original and owned by the authors.

Decreased bowel wall enhancement was the most specific indicator of bowel ischemia (specificity 100%, PPV 100%), consistent with previous reports [9]. However, its low sensitivity (26.7%) limits its utility as a standalone screening tool. A more significant finding of this study is the diagnostic value of Extravasation Type 2, defined as active contrast extravasation tracking along the bowel contour. In our multivariable analysis, this finding was an independent predictor associated with a significant increase in the risk of bowel ischemia. Anatomically, this pattern corresponds to distal mesenteric injuries involving the vasa recta. Current radiologic guidelines describe it as analogous to “bucket-handle tears” or mesenteric avulsions, which are classified as high-grade injuries [14]. Unlike proximal injuries, where collateral flow from the rich vascular network preserves perfusion (Extravasation Type 1), distal injuries involve severance of vessels directly supplying the bowel wall, leading to rapid devascularization and ischemia (Figure 3). Thus, the presence of contrast tracking along the bowel contour should be regarded as a critical imaging warning sign that warrants careful consideration for surgical exploration within the overall clinical context. However, CT extravasation patterns should not be regarded as primary determinants for choosing surgical versus nonoperative management, but rather as adjunctive imaging findings that may assist clinical judgment. Furthermore, the wide confidence intervals observed for certain CT findings, such as decreased bowel wall enhancement, underscore the importance of using these radiological signs as part of a multi-faceted clinical assessment rather than as isolated absolute metrics.

The relatively high sensitivity of the dependent portion fluid supports its role as a negative predictor. Although it cannot independently determine the need for surgery, it may help exclude bowel ischemia in equivocal cases [15].

Conversely, distinct CT findings such as isolated mesenteric haziness and hematoma were significant negative predictors of bowel ischemia. This inverse association offers valuable clinical insight. Mesenteric haziness and localized hematomas typically result from venous bleeding or injury to smaller mesenteric vessels, with the major arterial supply remaining intact. Recent studies support this finding, suggesting that focal mesenteric hematomas (typically <5 cm) without associated bowel wall thickening or peritoneal signs represent low-grade injuries that are highly amenable to conservative management [16]. In contrast, severe arterial injuries capable of causing ischemia often result in either the specific extravasation pattern described above (Extravasation Type 2) or thrombotic occlusion of the injured vessel [17]. In cases of occlusion, bleeding may be minimal or absent, preventing the formation of a significant hematoma. Therefore, a prominent mesenteric hematoma, in the absence of other ominous signs, may paradoxically reassure the clinician of preserved arterial inflow. Nevertheless, it is critical to emphasize that the inverse association of mesenteric haziness or hematoma with ischemia should not be misinterpreted as a ‘protective’ factor. These findings do not guarantee the absence of bowel injury and should not lead to a false sense of security; continued clinical vigilance remains mandatory as delayed complications can still occur.

Several previous studies have proposed CT-based scoring systems to aid in the detection of significant blunt bowel and mesenteric injuries and to reduce delays in operative intervention. These systems primarily incorporate findings such as free fluid, bowel wall thickening, mesenteric hematoma, or reduced bowel wall enhancement, with the goal of identifying injuries that warrant immediate surgical exploration. While these approaches have improved the early recognition of surgically significant injuries, they are not specifically designed to predict the subsequent development of bowel ischemia in patients who are initially hemodynamically stable [10,11].

In contrast, the present study focuses on early risk stratification for delayed bowel ischemia based on the initial CT findings obtained at presentation. In particular, the identification of contrast extravasation tracking along the bowel contour (extravasation type 2) highlights a distinct injury mechanism involving distal mesenteric vessels, which may not be adequately captured by existing grading or scoring systems. This distinction may explain why bowel ischemia can occur even in patients with relatively low-grade mesenteric injuries and underscores the complementary role of the CT predictors identified in this study.

These results imply that the interpretation of initial CT scans should extend beyond the simple presence of fluid. Clinicians should closely examine the pattern of contrast extravasation. However, it is crucial to emphasize that the absence of contrast tracking along the bowel contour does not guarantee safety, particularly when the initial CT is delayed. Hemorrhage tends to track along the bowel contour, producing the characteristic contrast-tracking appearance on CT. This finding, however, is transient. As the delay before CT increases, contrast disperses into the intraperitoneal cavity, and the pattern may disappear [18,19,20]. Consequently, in clinical practice, if the initial CT is obtained with a significant delay (e.g., >3 h), the specific extravasation pattern may be absent even in the presence of a bowel-adjacent mesenteric injury. Therefore, for patients with delayed presentation, the absence of extravasation should not be interpreted as definitive evidence of bowel viability, and the decision to proceed with NOM must be made with extreme caution.

This study has several limitations. First, as a single-center retrospective cohort review, findings are subject to selection bias. Second, the sample size was relatively small, reducing statistical power for rare findings. Third, although delayed scans were excluded to control for timing, the exact interval still varied, which could influence the detectability of transient signs. Fourth, the long study duration from 2013 to 2024 might introduce potential bias from subtle shifts in management strategies or scanner technology, although we attempted to mitigate this through standardized protocols. Additionally, the relatively small sample size for certain rare CT findings resulted in wide confidence intervals, which limits the definitive clinical application of those specific odds ratios. Finally, interobserver variability in interpreting subtle CT findings remains a potential confounder. To address these limitations, prospective multicenter studies are warranted to validate our results. In addition, the development of standardized CT scoring systems and the integration of AI-based image interpretation may further enhance early detection of bowel ischemia [21,22,23,24].

## 5. Conclusions

Specific CT findings on initial imaging may assist in early risk stratification for bowel ischemia after mesenteric injury following blunt abdominal trauma. While decreased bowel wall enhancement reflects established ischemia, contrast extravasation tracking along the bowel contour may be considered a significant risk factor that warrants careful clinical correlation.

In contrast, mesenteric haziness and mesenteric hematoma showed a lower association with delayed bowel ischemia in the present cohort; however, these findings must not be viewed as evidence of safety, and surgical consideration should always prioritize the patient’s overall clinical and hemodynamic status. In addition, larger prospective studies are warranted to validate the predictive value of specific contrast extravasation patterns and to better define their role in clinical decision-making.

## Figures and Tables

**Table 1:** Baseline characteristics of patients with blunt mesenteric injury.

Variable	Overall (*n* = 174)	Ischemia (*n* = 30)	Non-Ischemia (*n* = 144)	*p*-Value
Age (years)	53.70 ± 15.57	52.47 ± 14.26	53.96 ± 15.87	0.634
Male sex (%)	79.3%	86.7%	77.8%	0.331
ISS	13.78 ± 8.75	18.50 ± 9.22	12.80 ± 8.35	0.001 *
Shock Index ^†^	0.82 ± 0.28	0.84 ± 0.38	0.82 ± 0.26	0.675
RBC transfusion for 24 h (units)	2.36 ± 3.15	3.00 ± 3.10	2.22 ± 3.16	0.220
Cause of trauma				
In-car TA	73	16	57	0.222
Motorcycle TA	27	5	22	0.787
Pedestrian	20	0	20	0.027 *
Crush injury	32	8	24	0.203
Fall	12	0	12	0.225
Slip down	5	0	5	0.589
Bicycle	5	1	4	1.000

^†^ Shock index was calculated as heart rate (beats/min) divided by systolic blood pressure (mmHg). * Statistically significant values are indicated by *p* < 0.05.

**Table 2:** Univariate association between CT findings and bowel ischemia.

CT Finding	Ischemia (*n* = 30), *n* (%)	Non-Ischemia (*n* = 144), *n* (%)	Sensitivity (%)	Specificity (%)	PPV (%)	NPV (%)	OR (95% CI)	*p*-Value
Decreased bowel wall enhancement	8 (26.7)	0 (0.0)	26.7	100.0	100.0	86.7	109.18 (6.09–1957.81)	<0.001 *
Extravasation type 1 (Free intraperitoneal)	1 (3.3)	19 (13.2)	3.3	86.8	5.0	81.2	0.33 (0.06–1.81)	0.205
Extravasation type 2 (Contrast tracking bowel contour)	7 (23.3)	4 (2.8)	23.3	97.2	63.6	85.9	9.96 (2.86–34.70)	<0.001 *
Dependent portion fluid	21 (70.0)	67 (46.5)	70.0	53.5	23.9	89.5	2.60 (1.13–5.96)	0.026
Mesenteric haziness	5 (16.7)	66 (45.8)	16.7	54.2	7.0	75.7	0.25 (0.10–0.68)	0.004
Mesenteric hematoma	3 (10.0)	54 (37.5)	10.0	62.5	5.3	76.9	0.21 (0.07–0.68)	0.003
Pseudoaneurysm	10 (33.3)	61 (42.4)	33.3	57.6	14.1	80.6	0.70 (0.31–1.57)	0.418
Interloop fluid	10 (33.3)	39 (27.1)	33.3	72.9	20.4	84.0	1.37 (0.60–3.13)	0.508

* Statistically significant values are indicated by *p* < 0.05.

**Table 3:** Multivariable ridge-penalized logistic regression for bowel ischemia.

CT Finding	aOR (95% CI)	*p*-Value
Decreased bowel wall enhancement	5.45 (2.62–9.37)	<0.001 *
Extravasation type 1 (free intraperitoneal)	0.55 (0.32–1.01)	0.067
Extravasation type 2 (contrast tracking bowel contour)	3.07 (1.51–5.73)	<0.001 *
Dependent portion fluid	1.08 (0.70–1.91)	0.767
Mesenteric haziness	0.32 (0.19–0.57)	<0.001 *
Mesenteric hematoma	0.34 (0.20–0.53)	<0.001 *
Pseudoaneurysm	1.02 (0.55–1.90)	0.950
Interloop fluid	1.29 (0.68–2.06)	0.383

* Statistically significant values are indicated by *p* < 0.05.

## Data Availability

The data presented in this study are available on request from the corresponding author due to privacy/ethical restrictions.
